# West Nile Virus Encephalitis in a Patient with Neuroendocrine Carcinoma

**DOI:** 10.1155/2016/9497075

**Published:** 2016-07-19

**Authors:** Romina Deldar, Derek Thomas, Anna Maria Storniolo

**Affiliations:** Division of Hematology & Oncology, Indiana University School of Medicine, Indianapolis, IN 46202, USA

## Abstract

*Importance*. Oftentimes, when patients with metastatic cancer present with acute encephalopathy, it is suspected to be secondary to their underlying malignancy. However, there are multiple causes of delirium such as central nervous system (CNS) infections, electrolyte abnormalities, and drug adverse reactions. Because West Nile Virus (WNV) neuroinvasive disease has a high mortality rate in immunosuppressed patients, a high index of suspicion is required in patients who present with fever, altered mental status, and other neurological symptoms.* Observations*. Our case report details a single patient with brain metastases who presented with unexplained fever, encephalopathy, and new-onset tremors. Initially, it was assumed that his symptoms were due to his underlying malignancy or seizures. However, because his unexplained fevers persisted, lumbar puncture was pursued. Cerebrospinal fluid analysis included WNV polymerase chain reaction and serologies were ordered which eventually led to diagnosis of WNV encephalitis.* Conclusions and Relevance*. Patients with metastatic cancer who present with encephalopathy are often evaluated with assumption that malignancy is the underlying etiology. This can lead to delays in diagnosis and possible mistreatment. Our case highlights the importance of maintaining a broad differential diagnosis and an important diagnostic consideration of WNV encephalitis in patients with cancer.

## 1. Case Report

A 58-year-old male presented in the summertime with fever, generalized weakness, and encephalopathy after being found unconscious. Upon arrival to the hospital, he was disoriented to place and time, dysarthric, and amnestic to details of the event. Review of systems was positive for one week of progressive gait instability but negative for any other preceding or ongoing infectious or neurologic symptoms. He had no recent travel or exposure to sick contacts.

Seven months prior to his presentation, the patient had been diagnosed with a poorly differentiated neuroendocrine carcinoma, which presented primarily in his parotid gland. He had completed seven cycles of chemotherapy, with the last cycle given two weeks previously. Two months earlier, he had developed numerous asymptomatic brain metastases. A whole-brain radiation therapy was recommended for him; however, he declined. Other chronic medical problems included a history of prostate cancer in remission, dyslipidemia, and hypertension. He also had a history of “visual seizures” for which he was taking daily antiepileptic medication.

On presentation, the patient was somnolent but arousable. His vital signs revealed that he was febrile (temperature of 102.4 F) and tachycardic (heart rate of 126), blood pressure was 150/105 mmHg, respiratory rate was 14, and oxygen saturation was 95% on 2 liters per minute nasal cannula. Cardiopulmonary and gastrointestinal examinations were otherwise unremarkable. On neurological examination, the patient exhibited a resting tremor in both upper extremities, which his family reported was new within the past few days. There was no evidence of tongue biting, urinary incontinence, photophobia, or nuchal rigidity. Muscle strength, tone, and deep tendon reflexes were normal and symmetric. Babinski signs were absent. Sensory examination was normal.

Laboratory evaluation was significant only for mild hyponatremia (132 mmol/L) and elevated creatinine kinase (236 mmol/L). Tests for liver function, complete blood count, and urinalysis were unremarkable. Blood cultures and urine cultures were sent upon presentation and eventually revealed no growth. Urine drug screen was positive for cannabinoids only. Chest radiograph revealed no acute abnormalities. A noncontrasted head computed tomography was unremarkable with poor visualization of his known intracranial metastases. Initially, there was suspicion for an infectious etiology or progression of previously known brain metastases; therefore, the patient was started on empiric broad-spectrum antibiotics and systemic glucocorticoids for potential vasogenic edema until further diagnostic evaluation could be performed. His antiseizure medication was also continued.

One day after presentation, the patient's mental status was remarkably improved, although not completely to baseline. The transient nature of his encephalopathy raised suspicion of a generalized seizure as the inciting event with resultant temporary postictal state. Given this, an electroencephalogram (EEG) was obtained, revealing mild-to-moderate generalized background slowing, indicating diffuse encephalopathy, without any epileptogenic foci identified.

Two days after presentation, the patient remained febrile and tremulous; however, his cognition continued to improve. Because no definitive diagnosis had been reached and he continued to have unexplained fevers, a lumbar puncture was performed for further evaluation. Cerebrospinal fluid (CSF) studies revealed lymphocytic inflammation with an elevated glucose (84 mg/dL), elevated protein (89 mg/dL), and elevated total nucleated cell count (95/cumm), with a differential of neutrophils 8%, lymphocytes 53%, plasma cells 25%, and monocytes 14%. CSF Gram stain was negative, and cytology was negative for malignant cells. Herpes Simplex Virus-1 (HSV-1) and HSV-2 reverse transcription-polymerase chain reaction (RT-PCR) tests were negative, as was* Cryptococcus neoformans* antigen. CSF and serologic testing for West Nile Virus (WNV) IgM, IgG, and PCR was also ordered.

Approximately one week after presentation, a final diagnosis of acute WNV encephalitis was made. Although CSF IgG and PCR for WNV were negative, the patient's serum and CSF WNV IgM were qualitatively positive, establishing the diagnosis of WNV neuroinvasive disease [5, 8]. Empiric antibiotics and steroids were discontinued. Brain magnetic resonance imaging was obtained, which revealed stable intracranial metastases compared to one month earlier. Over a period of several days, his mental status improved completely to baseline and his fever and tremors subsided. He did, however, continue to have significant diffuse weakness, presumably secondary to prolonged hospital stay and systemic glucocorticoids. The patient was eventually discharged to a subacute rehabilitation facility.

## 2. Discussion

Oftentimes, when patients with metastatic cancer present with acute encephalopathy, it is suspected to be secondary to their underlying malignancy. Supporting this rationale is the fact that brain metastases affect 20 to 40 percent of cancer patients who are hospitalized, and neurologic complications are one of the most common reasons for their hospitalization [9]. Nevertheless, other causes of delirium, specifically central nervous system (CNS) infections, electrolyte abnormalities, vitamin deficiencies, seizures, and drug adverse effects, should be considered on initial evaluation. In a retrospective review by De la Cruz et al., opioid-related delirium accounted for 47.31% of missed delirium cases in cancer patients [10]. Primary CNS infection warrants specific consideration in the workup of delirium in cancer patients noting that 16% of patients with CNS infections have primary CNS tumors [11].  Table 1 lists infectious and noninfectious causes of meningoencephalitis worldwide that may affect immunocompromised patients, including cancer patients [11].

Although uncommon in the healthy population, WNV has an increased incidence in immunocompromised patients [12]. WNV can be a difficult diagnosis to make. It requires a high index of suspicion as presenting symptoms are nonspecific and initial brain imaging may reveal no abnormalities [12]. The presentation of WNV infection can vary from asymptomatic to a mild, febrile flu-like syndrome to more severe neurological symptoms. Less than 1% of individuals develop neuroinvasive disease, which manifests as meningitis, encephalitis, or polio-like flaccid paralysis [13]. Symptoms such as tremors, myoclonus, or extrapyramidal symptoms may be seen. Immunosuppressed patients, such as those receiving chemotherapy, are at higher risk for neuroinvasive disease [12]. Mortality rate can reach as high as 20% in these populations [14]. Treatments with interferon, intravenous immunoglobulin, and ribavirin have been used but lack high efficacy. Therefore, only supportive measures are recommended in most cases.

In our patient, workups of systemic infection, including blood cultures, urine cultures, and chest imaging, were all negative. CSF evaluation was pursued only after the patient's mental status and fevers did not improve with broad-spectrum antibiotics. Although CSF IgG and PCR for WNV were negative, this is common in acute WNV infection, given their low sensitivity [5]. Both serum and CSF IgM were positive, which is the gold standard for diagnosis of acute WNV encephalitis [15]. WNV-specific IgM antibodies are detectable 3 to 8 days after onset of illness and typically persist up to 90 days, whereas WNV-specific IgG is detectable after 7 days of illness onset and persists indefinitely [8, 16]. A positive IgM result combined with a negative IgG result suggests acute infection [16].  Table 2 illustrates the sensitivities and specificities of various diagnostic tests for WNV infection.

As described above, patients with metastatic cancer who present with encephalopathy are often evaluated with the assumption that malignancy is the underlying etiology. This can lead to delays in diagnosis and possible mistreatment. To date, there have been few case reports detailing the course of WNV infection in patients with malignancy. Our case highlights the importance of maintaining a broad differential diagnosis as well as a relatively rare but important diagnostic consideration of WNV encephalitis in this patient population. Because of its high mortality rate in immunosuppressed patients, timely diagnosis of WNV neuroinvasive disease should be made to ensure proper monitoring and supportive measures. It is therefore critical that multiple etiologies be considered in the workup of acute delirium in the patient with malignancy as determining the underlying diagnosis may obviate unnecessary diagnostic interventions and empiric treatments that could have adverse effects.

## Figures and Tables

**Table 1:** Infectious and noninfectious causes of encephalitis.

Infectious causes	Diagnostic tests (CSF, unless indicated)	Noninfectious causes
*Viruses*		
Enteroviruses	PCR	Opiates
HSV-1, HSV-2	PCR	Adverse drug reactions
VZV	PCR, virus-specific antibody	ADEM
EBV	PCR	CNS vasculitis
HIV	Western Blot, ELISA (blood)	
WNV	Virus-specific IgM	
TBEV	PCR	
JEV	Virus-specific IgM	
*Parasites*		
*Toxoplasma gondii*	PCR, culture	
*Trypanosoma cruzi*	PCR, culture	
*Fungi*		
*Cryptococcus neoformans*	India ink, cryptococcal antigen	
*Histoplasma capsulatum*	Serum: CSF antibody	
*Candida albicans*	Culture	
*Bacteria*		
MRSA	Culture	
*Streptococcus*	Culture	

HSV, Herpes Simplex Virus; VZV, Varicella Zoster Virus; EBV, Epstein-Barr Virus; HIV, Human Immunodeficiency Virus; WNV, West Nile Virus; TBEV, tick-borne encephalitis virus; JEV, Japanese encephalitis virus; MRSA, methicillin-resistant *Staphylococcus pneumonia*; CSF, cerebrospinal fluid; PCR, polymerase chain reaction; ELISA, enzyme-linked immunosorbent assay; ADEM, Acute Disseminated Encephalomyelitis; CNS, central nervous system.

References: [1–4].

**Table 2:** Diagnostic tests for acute West Nile Virus infection.

Test	Sensitivity	Specificity
PCR (serum)	10%	100%
PCR (CSF)	55%	100%
WNV-specific IgM (serum)	95%	90%
WNV-specific IgM (CSF)	95%	92%

PCR, polymerase chain reaction; CSF, cerebrospinal fluid; WNV, West Nile Virus.

References: [1, 5–7].
